# Our Duty to Our Patients, the People at Large, and to Our Profession

**Published:** 1862-07

**Authors:** W. H. Atkinson


### DENTAL REGISTER OF THE WEST.

VOL. XVI.] JULY, 1862. [No. 7.

### Original Essays and Communications.

### OUR DUTY TO OUR PATIENTS, THE PEOPLE AT LARGE, AND TO OUR PROFESSION.

BY DR. W. H. ATKINSON.

Read before the N. Y. Society of Dental Surgeons, on the evening of June 18, 1862.

THIS is a wide scope for activity for all our powers, and involves all the possible duties of life, outside of our own-selves. But we have the consolation of knowing that if all these duties to others be fulfilled, we incidentally secure complete performance of all that pertains to self. For to be useful to others demands of us the implicit obedience to all personal duties, that we may be in condition to do our duties to others at all times. I am aware that the proposition is equally true if viewed from the individual point of observation, and stated thus: If we fulfill all duties to ourselves, we shall secure the complete fulfillment of all duties to all others.

In this sense there can be no difference among us as to the general principle: But as to specific performance, duties become as various as the individuals of the race. Therefore, for general purposes, we must state broad principles, and for specific performance we must deal more in the detail of matters. For generalizations, we can not go beyond the simplicity and completeness of the Golden Rule; but for the application of this wholesome precept, we must become conversant with the facts and limitations of departments out of which individual duties take their origin and significance.

What, therefore, is our duty to our patient when first called upon to exercise our professional ability? Ans. Doubtless, the first duty is a careful survey of the case presented, that a correct and just diagnosis may be arrived at. And the next is a clear and candid statement of what may fairly be hoped for as the result of the best that can be done for him in the premises. It will be perceived that this implies a due preparation physically, mentally, morally and professionally on our part, before placing ourselves in circumstances to be consulted, so that we may avoid the humiliation and other evils consequent upon a premature assumption of the delicate and difficult exercise.

When we have clearly made out the case, and it so happens that it is a difficult one, say for example, one for regulating a bad case of irregularity, we should divest ourselves of all longings for reputation or remuneration so far as honestly to settle in our own minds whether there may not be a neighboring professional brother who can render the applicant a better service than we can, and if this be our conviction, the course to pursue is plain. But if we feel as capable of rendering a good service as any in the range of our acquaintance, the course is also plain, which is to begin just right, continue right, and then we are sure to come out just right, and happily bring the matter to the right issue.

It is the right of every member of every profession to request the counsel of any other member of the body, provided it be done in proper time and place; and in no case is the member so consulted at liberty to shuffle out of the responsibilities of the case so presented, but he is bound by the laws of God and humanity, if not by his apprehension of professional etiquette, to take it carefully and earnestly into consideration, and patiently go over the details, that he may afford the help desired to the honest inquirer. After this is all kindly and fraternally done, it is at the option of the one consulted to accept a remuneration or not, as he is disposed.

Thus intimately are all our duties to people, patients and our brethren blended and interblended, so that our only safety is in acting above the groveling ignorance of selfishness, in all that we do to any and to all. So shall we be in the way of understanding each advance made by ourselves and others, through the frequent comparisons of notes, by frank consultations upon all anomalous and difficult cases, whether of diagnosis or performance. And thus it shall soon become the rule to succeed, and the exception to fail or make a mistake, and thus reverse the old order of things, in which failures were frequent, and mistakes by no means strange occurrences among us!

Our duty to the people at large is to inform them of the consequences of infraction of the laws of being, and especially the laws of development and growth of the teeth. Much more of general bodily and mental, not to say moral, health depends upon sound dental organs than has ever been taken into the account, when trying to correct the errors of individual, family and neighborhood jars. "Good teeth, good digestion,—good digestion good disposition" of the child or adult is an aphorism that would well repay a close scrutiny and study. Dentists may do far more permanent good to the communities in which they live by diffusing correct principles that will prevent disease and pain, than by ever so long-continued and worthily bestowed effort of superior skill in correcting these errors, after they have once taken deep hold upon teeth and entire digestive system, thus disturbing moral and mental being to indefinite extent.

Our duty to our professional brethren is a more difficult subject to fully apprehend and live up to, because there is such difference of opinion of who are our brethren, or who constitute those who should be recognized as members of the profession.

The only safe way is to treat *all* with courteous and indly behavior, and being always ready to shed light upon all dark and vexed subjects pertaining to the practice, if for no other reason, because it will lessen the amount of mischief perpetrated upon the poor and the ignorant who seek relief at the hand of any who has the impudence to announce himself to be a member of our to be honorable body.

The peculiar condition of the science and art of dentistry at present is such that it is a difficult matter to give honor only to whom honor is due, and at the same time not to withhold it from many worthy of honorable mention and fraternal support.

There is undoubtedly much less excuse now for young mechanics and sharp handicraft men rushing into the practice of our difficult profession, without adequate study and preparation, than formerly; as there are now not wanting Dental Colleges and private tutors, competent and willing to instruct for reasonable compensation, which was not the case twenty-five or thirty years ago. Then it required a handsome sum to pay for the poor instruction and worse recipes then to be had. But thank God and a few earnest minds, we have outgrown the days of this ignorance and vile concupiscence! Any earnest and honest man may avail himself of the arduous labors of years of study and toil of the few good and true men who have in some sort codified, though loosely and fragmentarily, the principles and practice thus collated and put forth as a basis upon which to build an honorable and efficient system of dental practice, for a tithe of the cost to those who were forced, from the nature of the case, to come up through great tribulation of mingled blunders and successes.

It is manifestly our duty to sustain every member of the profession so far as we can, without thereby endorsing his errors, or seeming to wink at or coincide with malpractices in our body. But that which may now be tolerated as bearable practice will in a very few years become rankest disobedience to the laws governing us, if we will only carefully live up to the light we have. For it is notorious that many able men are giving countenance to the expediency of practices that they are frank to acknowledge are not the highest and best that they can conceive, or would practice under "other circumstances." We should control these "circumstances" more, and let them govern us less and less year by year—as indeed is now happily becoming more and more the rule among us; chiefly because some have discharged the duties they owed to their professional brethren. Now, let us all go and do likewise, letting all the light possible shine in upon our darkness, until we shall all be full of light.

How may we best accomplish all these duties to patient, people and profession? Ans. By being heartily in earnest to do our best in every operation which we undertake; and only working for such persons and at such operations as are feasible—such patients as are compatible to us and we compatible to them—such operations as we feel ourselves complete masters of in diagnosis, prognosis and performance; and in laying aside all partiality for one operation as more honorable than another, or setting up one department of the profession as a merely mechanical appendage to the more dignified surgical department of our duty to patients. If surgical knowledge and skill were absent, where were the use of all the manipulative ability extant? And if this be wanting, what would all the mere science of the profession avail to the suffering patient?

Our profession is not complete without both surgical and mechanical skill in full possession and exercise, no more than the circulation can be healthy and sustaining in the absence of digestion in proper order, or in the absence of lungs or gills to receive the vital element from the atmosphere, and mingle it in the vital current in due proportion of admixture, to insure the necessary fluidity to find its way into and through the finest ramifications of the capillary system, where all these phenomena called vital take place.

Mouth and digestive tube in good working condition, and lungs, arterial, venous and nervous system in normal play, we may hope to have nutriment properly prepared and appropriated; so, professor and pupil in earnest and in harmony, we may hope to have intelligent members of our body; and surgical and mechanical knowledge and skill in due equipoise, we may hope to have a vigorous and harmonious profession.

